# The role of prefrontal catecholamines in attention and working memory

**DOI:** 10.3389/fncir.2014.00033

**Published:** 2014-04-08

**Authors:** Kelsey L. Clark, Behrad Noudoost

**Affiliations:** Department of Cell Biology and Neuroscience, Montana State UniversityBozeman, MT, USA

**Keywords:** dopamine, reward, top-down control, pathophysiology, frontal eye field, V4, extrastriate cortex

## Abstract

While much progress has been made in identifying the brain regions and neurochemical systems involved in the cognitive processes disrupted in mental illnesses, to date, the level of detail at which neurobiologists can describe the chain of events giving rise to cognitive functions is very rudimentary. Much of the intense interest in understanding cognitive functions is motivated by the hope that it might be possible to understand these complex functions at the level of neurons and neural circuits. Here, we review the current state of the literature regarding how modulations in catecholamine levels within the prefrontal cortex (PFC) alter the neuronal and behavioral correlates of cognitive functions, particularly attention and working memory.

## Introduction

Attention, working memory, impulse control, and other “top-down” cognitive functions have long been known to depend on the prefrontal cortex (PFC) (Ghent et al., 1962; Chao and Knight, 1998; D'Esposito and Postle, 1999). Many of these cognitive functions are disrupted in mental disorders such as attention deficit hyperactivity disorder (ADHD), Parkinson's disease, and schizophrenia. Studies in human and non-human primates have implicated prefrontal catecholamines in control of cognitive functions. Notably, drugs altering catecholamine signaling have been used to treat the symptoms of some of these mental illnesses. Consequently, an imbalance in prefrontal catecholamines has long been a suspected cause of the cognitive component of these mental illnesses. Our goal is to review studies examining the contribution of prefrontal catecholamines to cognitive tasks and their dysfunction. Due to known differences between rodents and primates (Berger et al., 1991), this review will be focused on studies in human and non-human primates. Among catecholamines, the main focus will be on dopamine (DA), however the role of norepinephrine (NE) will also be briefly addressed. We survey the evidence implicating prefrontal catecholamines as the neurochemical mediator of the neural and behavioral signatures of attention and working memory, and link these neurobiological findings to the etiology and treatment of cognitive impairments in mental disorders.

## Effects of DA within PFC

The importance of prefrontal DA in delayed-response tasks was established very early on (Brozoski et al., 1979), and much work has since gone into unraveling the details of this dependence (see Table 1). The PFC receives DA-ergic projections from both the ventral tegmental area (VTA) and the substantia nigra (Porrino and Goldman-Rakic, 1982; Levitt et al., 1984; Goldman-Rakic et al., 1992). DA neurons in the VTA and substantia nigra exhibit both tonic activity, and phasic responses associated with the expectation of reward (Schultz et al., 1993) or reward prediction errors (Schultz, 1998). While DA neurons are activated by the spatial cue in the working memory tasks discussed below (since it signals the availability of a reward in the near future), this activation differs from that observed in PFC itself in that it does not reflect the cue position, nor does it continue throughout the delay period (Schultz et al., 1993). Therefore, the incoming DA-ergic input to PFC does not directly encode the remembered stimulus, but could potentially serve to “tune” the prefrontal network for optimal activity.

**Table 1:**
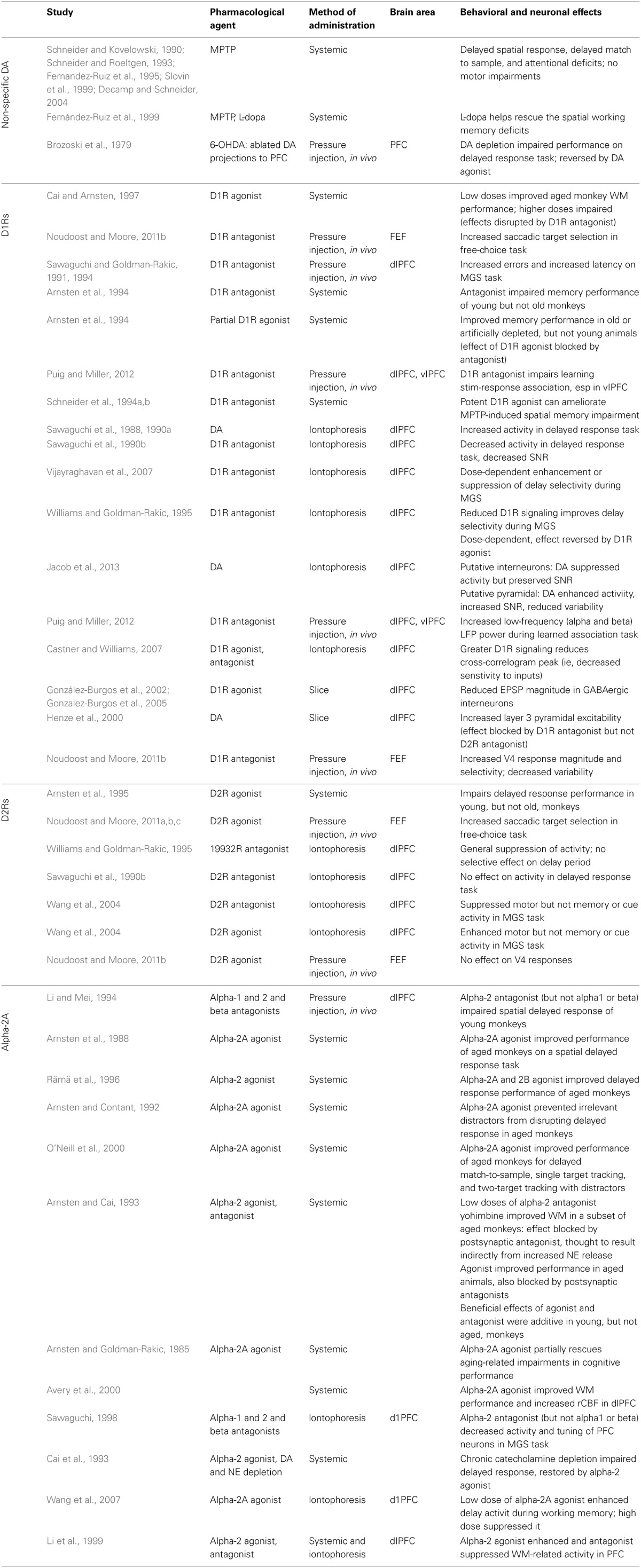
**Studies examining the contribution of prefrontal catecholamines to the behavioral and neural correlates of working memory in non-human primates**.

Studies are divided by neuromodulator (dopamine or norepinephrine) and specific receptor (D1R, D2R, alpha-2A) where applicable. Abbreviations: MPTP, 1-methyl-4-phenyl-1,2,3,6-tetrahydropyridine, which kills dopaminergic neurons in the substantia nigra; 5-OHDA, 5-hydroxydopamine, which selectively kills dopaminergic and noradrenergic neurons; FEF, Frontal Eye Field; dlPFC, dorsolateral prefrontal cortex; vlPFC, ventrolateral prefrontal cortex; SNR, signal-to-noise ratio; MGS, memory-guided saccade.

**Figure 1:**
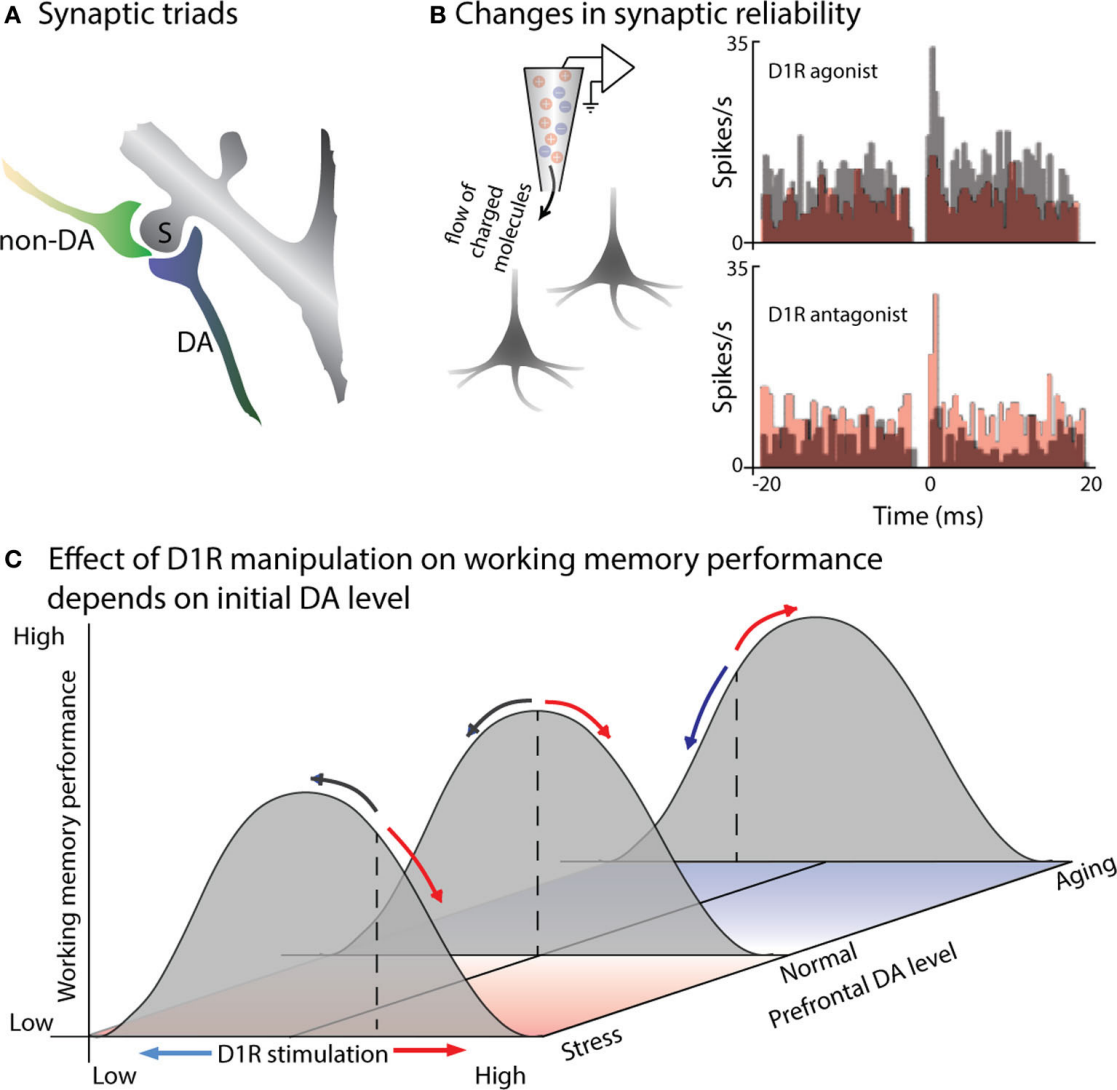
**DA modulates the efficacy of synaptic connections within PFC. (A)** Cartoon illustrates a characteristic “synaptic triad” in monkey PFC: the same spine (S) is postsynaptic to both a DA-positive axon (DA) and a non-immunoreactive axon terminal (non-DA). The synapse of the DA axon is symmetric, while the non-DA axon forms an asymmetric synapse. Based on electron micrography and immunostaining of synaptic connections in layer II of monkey PFC (from Goldman-Rakic et al., 1989). **(B)** D1R signaling alters synaptic efficacy, a measured by changes in the synchronous firing of pairs of PFC neurons. Castner and Williams (2007) simultaneously recorded from pairs of neurons in monkey PFC before and after delivering a D1R agonist or antagonist using *in vivo* iontophoresis. Cross-correlograms depict the number of spikes occurring at a particular time-lag relative to spikes of the other neuron in the pair: a peak at 0 ms indicates simultaneous firing due to common input. The top pair of PFC neurons exhibited a 0 ms peak prior to drug infusion (gray): application of a D1R agonist eliminated this peak (red), presumably by disrupting the efficacy of common input. In the bottom plot, the neurons did not show evidence of common input during the control recordings (gray), but a peak emerged following application of a D1R antagonist (red), reflecting stronger or more reliable excitation from their common inputs. **(C)** Effects of D1R agonists and antagonists on working memory performance will depend on initial levels of PFC DA. Gray curves illustrate working memory performance as a function of PFC DA level: performance is greatest at an intermediate level, with insufficient or excessive DA leading to impaired performance. Basal DA levels (illustrated by the dashed lines) are usually tuned for optimal performance (middle curve), but are sub-optimal in aged animals (back curve), and above optimal in the case of stress (front curve). D1R agonists (red arrows) and antagonists (blue arrows) effect working memory performance differently based on initial DA levels: if initial DA levels are supra-optimal, as in stress, then D1R antagonists will move DA signaling toward the optimal level, improving performance, while agonists will further impair performance. If initial DA levels are below optimal, as in aged animals, then D1R agonists will increase DA signaling back toward optimal levels, improving performance; D1R antagonists will move DA levels further from optimal, impairing performance.

In order to understand the effects of prefrontal DA release on neural activity, first let us consider DA receptors and the anatomy of DA-ergic terminals in PFC. DA receptors are G-protein-coupled receptors, modulating neuronal activity via intracellular signaling cascades rather than directly inducing either excitatory or inhibitory postsynaptic currents (Yang and Seamans, 1996; Lachowicz and Sibley, 1997; Missale et al., 1998). The five types of DA receptor are commonly divided into two classes: the D1 family (comprised of D1 and D5 receptors) and the D2 family (D2, D3, and D4 receptors) (Missale et al., 1998; Seamans and Yang, 2004). Expression for D1 receptors (D1Rs) is enriched in the PFC of both primates and rodents, suggesting an important role in specifically prefrontal circuit functions (Lidow et al., 1991; Goldman-Rakic et al., 1992). Within PFC, D1Rs are expressed in both superficial and deep cortical layers, while expression of the less abundant D2Rs is limited to the infragranular layers (Lidow et al., 1991). Although this bilaminar distribution pattern is evident at birth, layer three undergoes a dramatic post-natal increase in the density of DA innervation, which is then subject to layer-specific remodeling and decreases in DA axon density during adolescence (Lewis and Harris, 1991; Rosenberg and Lewis, 1995; Lewis, 1997); during this period performance on delayed response tasks improves and becomes more dependent on the PFC (Alexander and Goldman, 1978). Goldman-Rakic and colleagues used immunohistological staining, Golgi impregnation and electron microscopy to examine DA-ergic synapses in the PFC. They found that DA-ergic boutons were part of synaptic triads, in which the DA-positive bouton formed a symmetric synapse, while an unlabeled asymmetric synapse (of the type associated with excitatory inputs) contacted the same dendritic spine (Figure 1A). Many of the postsynaptic neurons appear to be pyramidal cells. However, targets of DA-ergic projections include both pyramidal cells and fast-spiking interneurons (Goldman-Rakic et al., 1989; Sesack et al., 1995, 1998). D1Rs can also be located outside of synapses (Smiley et al., 1994), suggesting at least some slow timecourse effects as DA diffuses to these more remote sites of action. Some DA axonal varicosities also appear to be localized outside of synaptic specializations (Smiley and Goldman-Rakic, 1993), and may contribute to extrasynaptic “volume transmission” effects (Zoli et al., 1998). This anatomy seems conducive to dopamine playing a modulatory role, regulating the efficacy or strength of prefrontal signals originating elsewhere.

The role of dopamine in modulating glutamatergic activity, suggested by the presence of synaptic triads, has been directly tested in slices using dual whole-cell patch clamp recordings, examining the effect of DA application on synaptic transmission between neurons. These experiments revealed that DA reduces the reliability of excitatory neurotransmission by reducing the probability of glutamate release presynaptically (Gao et al., 2001). Consistent with this finding, the consequences of this reduced reliability of synaptic transmission can be read out in the synchronous activity of neighboring prefrontal neurons *in vivo* (Figure 1B): iontophoretic application of a D1R antagonist introduces a peak in the cross-correlogram between prefrontal pyramidal neurons, as the reliability of transmission from mutual inputs increases (Castner and Williams, 2007). Conversely, iontophoretic application of a D1R agonist decreases synaptic efficacy, disrupting common excitatory input and eliminate existing cross-correlogram peaks for neighboring prefrontal neurons. Interestingly, DA may have different direct effects on excitatory and inhibitory neurons within PFC (Gao and Goldman-Rakic, 2003; Jacob et al., 2013). Using iontophoresis to examine the effects of DA on prefrontal visual responses during a visual detection task, Jacob and colleagues found two distinct types of DA-ergic modulation (Jacob et al., 2013) One group of PFC neurons, which included all the modulated narrow-spiking, putatively inhibitory neurons, was inhibited by DA; these showed short onset latency of DA effects (~10 ms), with no change in signal-to-noise ratio (SNR) or inter-trial variability. A second set of prefrontal neurons was excited by DA application, displaying an increase in SNR and decrease in inter-trial variability; this effect was slower (~200 ms) and observed only in broad-spiking, putatively pyramidal neurons. These direct effects of DA on the excitability of individual neurons of different types will then interact at the population level—for example, the activity of inhibitory neurons helps shape the tuning of excitatory neurons during working memory (Rao et al., 2000; Constantinidis et al., 2002).

In addition to modulating neural activity within PFC, DA also alters activity-dependent plasticity (Gurden et al., 2000; Pawlak and Kerr, 2008; Zhang et al., 2009). Such plasticity has long been the focus of study in relation to addiction; however, these changes may also play a more general role in associative learning. Recent work in rodent PFC suggests that both D1R and D2R signaling contribute to changes in plasticity, with D2R actions on inhibitory interneurons gating potentiation, and D1Rs on postsynaptic neurons controlling the size of the temporal window during which coincident spikes induce potentiation (Xu and Yao, 2010). These effects of DA on plasticity, and the known firing of DA inputs to PFC in response to prediction errors, suggest an influence of prefrontal DA on learning, and indeed a recent study reveals just such an effect (Puig and Miller, 2012). Local injection of a D1R antagonist into ventrolateral prefrontal cortex (vlPFC) was found to impair monkeys' acquisition of novel visuomotor associations, without impairing performance on familiar associations. This behavioral effect was accompanied by changes in prefrontal activity, again observed for novel but not familiar cues: selectivity of individual neurons for the upcoming motor response decreased, while synchronous discharge and low-frequency LFP power increased. Human experiments have also linked phasic activity in midbrain DA nuclei and PFC with context-dependent working memory performance (D'Ardenne et al., 2012). Thus, phasic discharge of prefrontal DA inputs may be particularly important during the learning of novel associations and tasks, or contextual switching between rules.

Williams and Goldman-Rakic used iontophoresis in a behaving monkey to extend these findings on the cellular effects of DA to its role in prefrontal circuits during a spatial working memory task (Williams and Goldman-Rakic, 1995). Iontophoretic application of a D1R antagonist during spatial working memory selectively enhances delay-period activity representing the remembered location. The effect is dose-dependent, with enhanced delay activity for an intermediate level of D1R antagonist, but suppression of both delay and visual activity in the same cell when a greater concentration of the drug is applied. This “inverted-U” dose-dependency, in which an intermediate level of DA signaling produces more selective memory activity, has also been observed for a D1R agonist: low doses suppressed only responses to non-preferred locations, enhancing the spatial tuning of delay activity, while higher doses suppressed activity altogether (Vijayraghavan et al., 2007). How to reconcile the apparently contradictory findings that a low dose of either a D1R agonist (Vijayraghavan et al., 2007) or a D1R antagonist (Williams and Goldman-Rakic, 1995) improves the selectivity of PFC delay activity? Presumably the answer lies in the original level of DA-ergic tone in the PFC neurons being studied (Figure 1C), although it remains unclear why these studies would have a systematic difference in baseline prefrontal DA-ergic stimulation. The known elevation of prefrontal DA by stress (Thierry et al., 1976; Roth et al., 1988; Abercrombie et al., 1989) further raises questions as to how the stresses affecting laboratory animals may impact their baseline DA-ergic tone, and thus the effects of pharmacological agents. Also note that the basis for improved delay selectivity appears to differ for the two agents: D1R antagonists may improve tuning by increasing the level of delay firing for the preferred location, while the D1R agonist selectively reduces firing for the non-preferred cue locations.

Several biologically-plausible neurocomputational models have been developed to incorporate DA or NE-ergic modulation of prefrontal activity (Servan-Schreiber et al., 1998; Durstewitz et al., 2000; Brunel and Wang, 2001; Chadderdon and Sporns, 2006; Eckhoff et al., 2009; Avery et al., 2013). One such model (Chadderdon and Sporns, 2006) of task-oriented behavioral selection incorporates such disparate brain regions as early visual areas, inferotemporal cortex, PFC, basal ganglia, and anterior cingulate cortex. At the heart of this model is a mechanism that simulates exogenously induced changes in prefrontal DA release, which is thought to underlie the updating and maintenance functions of working memory. More recently, Avery et al. (2013) constructed a model of PFC designed to capture the effects of signaling through both DA (D1) and NE (alpha-2A and alpha-1) receptors. Both of these models were able to reproduce the “inverted-U” effect of catecholamine signaling, with impaired working memory representations when levels were too high or too low. The former model incorporated changes in prefrontal DA levels over the course of a delayed match to sample task, using these changes to switch the prefrontal network between states of updating based on current inputs vs. maintaining previous inputs, while the latter instead examined the effects of tonic DA and NE tone on network behavior. The extent to which fluctuations in PFC DA levels during different task epochs occur or contribute to task performance remains experimentally unproven.

The effect of DA on the activity of prefrontal neurons is complicated, involving multiple mechanisms of direct and indirect action through D1Rs and D2Rs, affecting presynaptic release, NMDA, GABA, AMPA, Na^+^, Ca^2+^, and K^+^ currents, among others (Seamans and Yang, 2004). Various studies have reported either primarily inhibitory (e.g., Pirot et al., 1992), excitatory (e.g., Henze et al., 2000), or heterogenous (Jacob et al., 2013) effects of DA on PFC neurons. The main points we wish to emphasize here are that DA acts as a neuromodulator, altering the efficacy of synaptic input to prefrontal neurons, and that there is some optimal level of DA-ergic stimulation for a neuron to experience, with greater or lesser DA signaling leading to an erosion of task related activity.

## Dopamine, reward, and visual attention

Given the known firing of prefrontal DA afferents in response to reward expectation (Schultz, 2013), and the ability of expected reward to modulate responses throughout the brain, we cannot discuss the role of DA in prefrontal control of cognitive functions without considering the effects of reward and to what extent they can be separated from the other roles of prefrontal DA signaling. In the following sections we first discuss the difficulties in parsing the behavioral effects and neural signatures of attention and reward. We review the known role of both prefrontal DA and reward in modulating responses in visual cortex, and the evidence for and against prefrontal neurons receiving DA input themselves representing reward value. The evidence suggests that prefrontal DA contributes to both representations of target value and to the behavioral and neural signatures of attention, although further studies will be needed to determine if DA's roles in these processes are dissociable.

### Dissociating neural signatures of attention and reward

**Figure 2:**
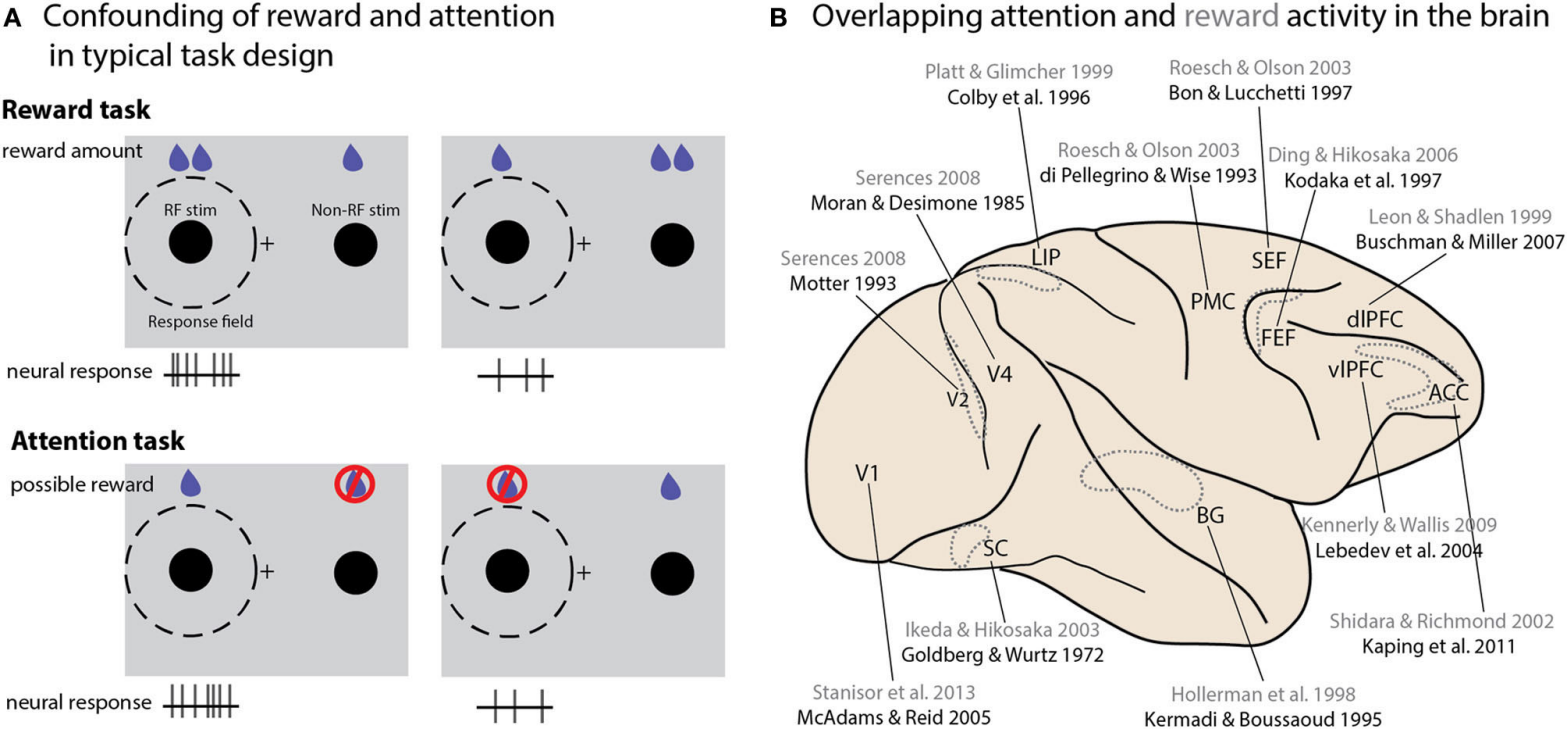
**Interactions between attention and reward. (A)** A schematic illustration of typical tasks used to study reward and attention, and how the differences in potential reward and neural activity are similar between the two paradigms. Consider two studies conducted in V1 (Stănişor et al., 2013 and McAdams and Reid, 2005). To study the effect of reward size in the Stănişor task (schematically illustrated in the top panel), two potential targets appear, with colors indicating different reward values. Neural activity recorded at this point in the task reflects the relative value of the target in the RF (higher activity when the RF target offered a greater reward than the non-RF target); a subsequent cue instructs the monkey which target to saccade to. In the McAdams and Reid attentional paradigm (bottom panel), a cue indicates which of two stimuli should be monitored for a change, which instructs an eye movement response to a separate location. Changes at the uncued location must be ignored, and will never lead to rewards. Neural activity is higher when the stimulus in the RF is cued. In both cases higher expected reward value for the stimulus in the RF is associated with greater neural activity. **(B)** An overview of brain areas in which neural activity reflecting both attentional modulation and reward value has been reported. Only a single study is cited for each area; reward studies are in gray, attention studies in black. Dotted outlines represent structures not located on the cortical surface, either within sulci or deeper within the brain. Abbreviations: PMC, premotor cortex; vlPFC, ventrolateral prefrontal cortex; dlPFC, dorsolateral prefrontal cortex; SC, superior colliculus; BG, basal ganglia; LIP, lateral intraparietal area; SEF, supplementary eye field; ACC, anterior cingulate cortex; FEF, frontal eye field.

DA release is associated with reward cues or expectation (Schultz, 2002). The involvement of DA signaling in both attention and reward raises the question of how these mechanisms overlap or diverge. Indeed, many behavioral tasks manipulate attention or reward in such a way that these two properties cannot truly be distinguished from one another (Maunsell, 2004). Consider a typical study seeking to identify a neural correlate of reward size (Figure 2A). One stimulus is placed within the neuron's response field (RF), a second outside it; the relative size of the reward associated with the two locations is then varied, either in blocks or from trial to trial based on some cue. A neuron that displays greater activity when the high-reward stimulus appears in its RF is typically reported as encoding reward expectation or value. The same logic applies to reward probability, although this manipulation must be done in blocks. Now consider a typical “attention” task: multiple stimuli appear onscreen, and one of them must be monitored for a behavioral response—again, the selected location may be held constant over a block of trials or varied from trial to trial based on a cue. Sometimes the task occurs only or more frequently at the cued stimulus—in other cases the animal is explicitly trained not to respond to changes at the uncued location; in either version of the attention task, reward is exclusively or predominantly associated with the stimulus at the attended location, and yet in these studies a difference in firing rate is attributed to the locus of attention rather than an expected reward. Conversely, the “reward” activity we described in the previous experimental design could be attributed to attentional modulation, given that on a behavioral level the expectation of a reward attracts attention (Posner, 1980). Importantly, many areas reflecting attentional modulation in their neural activity also exhibit reward-dependent activity (Figure 2B). A recent study of the effects of reward on activity in primary visual cortex showed that the strength of reward-size modulation across cells was strongly correlated with their modulation by attention, suggesting that the neural sources of these effects may be overlapping, if not identical (Stănişor et al., 2013, discussed further in the next section). Since this critique originally appeared a decade ago, many studies that experimentally manipulate reward values acknowledge the potential confound, or even explicitly attribute their findings to attention (Kennerley and Wallis, 2009), but few attempt to dissociate the two processes. Even studies using paradigms designed to differentiate representations of reward from general behavioral salience (Leathers and Olson, 2012) have proven controversial (Leathers and Olson, 2013; Newsome et al., 2013), or generated results that suggest reward cues can drive attentional allocation in ways that prove detrimental to task performance (Peck et al., 2009).

### Modulation of visual representations by PFC DA and reward

**Figure 3:**
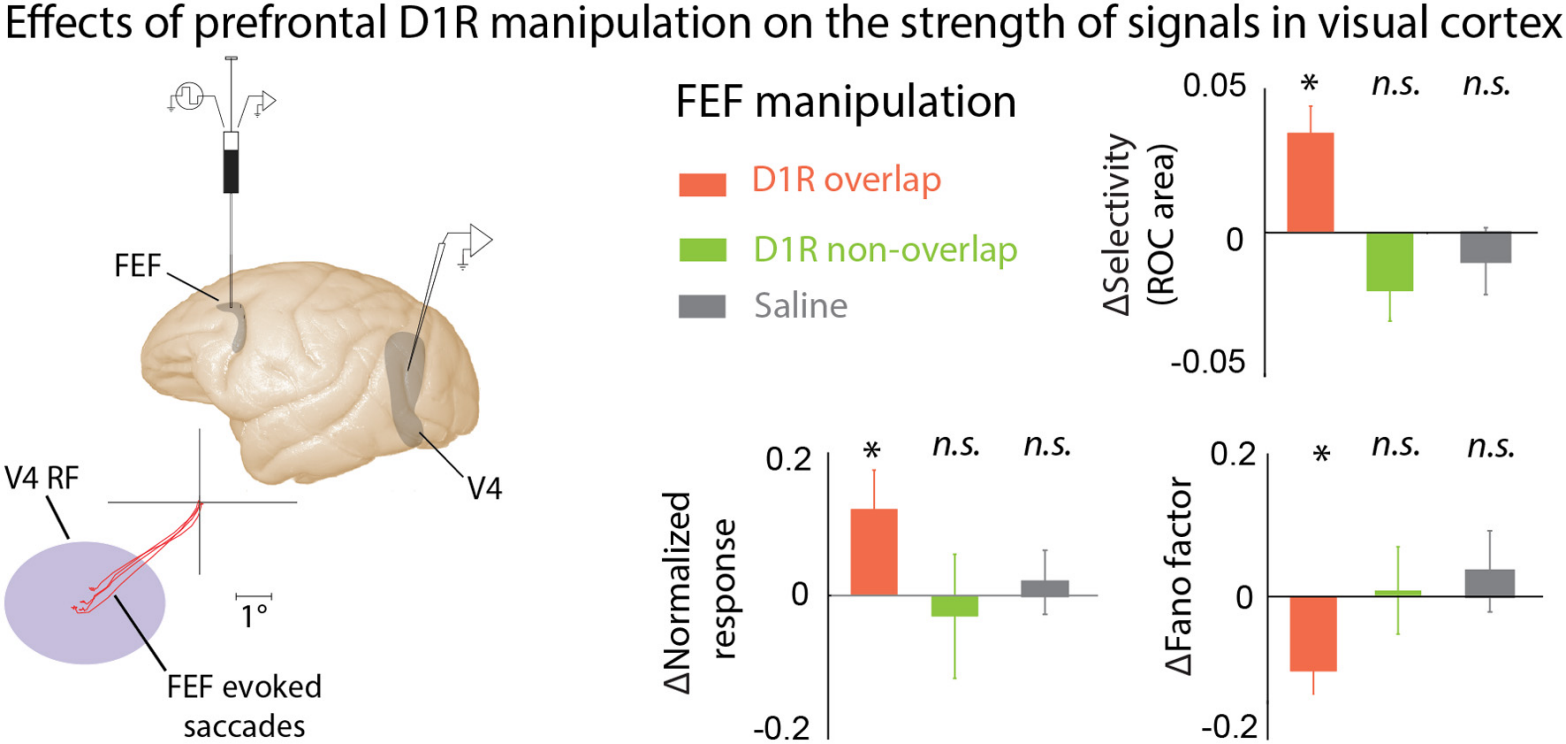
**The effects of PFC DA on visual cortical activity.** Manipulating D1R-mediated FEF activity enhances visual representations in area V4. Noudoost and Moore (2011b) infused a D1R antagonist into the FEF while recording from V4 neurons with RFs either overlapping or not overlapping the area of space represented at the site of drug infusion; the visual responses of the same V4 neurons were recorded before and after infusion of drugs into the FEF. FEF RF center was estimated based on the endpoints of microstimulation-evoked saccades. FEF D1R manipulation caused an increase in orientation selectivity, increase in response magnitude, and decrease in response variability at overlapping V4 sites (orange bars); no effect was seen for non-overlapping V4 sites (green), or saline infusions (gray). These changes in V4 responses with FEF D1R manipulation mimic those seen during covert attention. ^*^p <0.05.

Although many studies have examined the effect of DA-ergic agents on prefrontal activity, and prefrontal activity has long been believed to modulate responses in visual cortex during attention and working memory, until recently no one had directly examined the effect of locally manipulating prefrontal DA signaling on visual responses in other cortical areas. Noudoost and Moore (2011b) examined the long-range effects of altering prefrontal DA signaling on visual responses in extrastriate area V4. V4, like much of visual cortex, receives direct projections from the Frontal Eye Field (FEF) part of the PFC, an area strongly implicated in controlling spatial attention (Moore and Fallah, 2004; Armstrong et al., 2009; Clark et al., 2012), and it is believed that these projections may be the source of the changes in activity observed in V4 during the deployment of covert attention (Moore and Armstrong, 2003; Awh et al., 2006; Noudoost et al., 2010, 2014; Squire et al., 2013; Clark et al., 2014). Noudoost and Moore examined the effects of manipulating either D1Rs or D2Rs on V4 visual responses during a passive fixation task, and their effect on saccadic target selection in a free-choice task (Noudoost and Moore, 2011a,b). While both D1R and D2R manipulations increased the monkey's tendency to choose the saccade target in the affected region of space, biasing saccadic target selection, only D1Rs had an impact on V4 visual responses. Local injection of a D1R antagonist into the FEF enhanced the strength of visual signals in V4: response magnitude increased, orientation selectivity was enhanced, and trial-to-trial variability decreased (Figure 3). All of these changes are also observed in V4 when covert spatial attention is directed to the V4 neuron's RF (Moran and Desimone, 1985; McAdams and Maunsell, 2000; Reynolds et al., 2000; Mitchell et al., 2007). The reason for the differing effects of FEF D1R and D2R manipulations on V4 activity, but common effects on target selection, may lie in the patterns of receptor expression within the FEF. D1Rs are expressed in both the supragranular layers, which project to V4, and infragranular layers, which contain neurons projecting to motor areas such as the superior colliculus. In contrast, D2Rs are primarily expressed in the infragranular layers. This pattern of expression could account for both receptors influencing target selection, while only D1Rs alter V4 responses (Noudoost and Moore, 2011c).

Neurophysiological experiments in V1 have provided a direct comparison of the effects of attention and reward on visual cortical responses (Stănişor et al., 2013). Visual responses were shown to be modulated by the relative reward value of the RF stimulus; moreover, the magnitude of this modulation was strongly correlated with the strength of atttentional modulation during a later time window in the same task, and the onset latencies of the two effects were indistinguishable. Like attentional modulation, the neural effects of reward value were dramatically enhanced in the presence of a second stimulus. Human fMRI experiments have also demonstrated a D1R-dependent reward modulation of visual cortical activity (Arsenault et al., 2013). These effects of reward on visual cortex may not be attributable to the PFC—they could result from a bottom-up influence of DA-ergic changes in LGN signaling (Zhao et al., 2002), or via direct DA release from midbrain projections (Lewis et al., 1987). However, several aspects of the findings argue in favor of a prefrontal origin to these effects: the strong correlation with attention in the Stănişor case, the presence of this modulation even in trials without a visual stimulus in the Arsenault paper, the lower density of DA-ergic projections to visual cortex (Berger et al., 1988), and the proven ability of DA-ergic PFC activity to modulate representations in visual cortex (Noudoost and Moore, 2011b), make PFC a likely source of this reward-induced modulation.

### Representation of reward value by PFC neurons and the role of DA in this representation

Multiple studies have looked for representations of reward value in PFC. Leon and Shadlen (1999) examined the effect of centrally cued reward size on FEF and dlPFC responses during a memory-guided saccade task. They found an effect on reward size on responses in dlPFC, but not FEF; this dlPFC reward-size dependent activity continued throughout the delay period. Interestingly, the presence of reward-size information in dlPFC responses was dependent on the simultaneous maintenance of a spatial memory: in a variant of the task in which the reward cue appeared before the spatial cue, no reward size information was present until after the subsequent spatial cue appeared. However, findings by Ding and Hikosaka suggest that the FEF will also represent reward size information under certain conditions: specifically when the reward is tied to a particular location (Ding and Hikosaka, 2006). Using an asymmetrically rewarded memory-guided saccade paradigm, in which the relative value of the two target locations varied between blocks of trials, they found that about 1/3 of FEF neurons were selective for the location of the larger reward during the cue period. This may reflect the stronger retinotopic organization of the FEF in comparison to dlPFC (Suzuki and Azuma, 1983; Bruce et al., 1985; Funahashi et al., 1989). Interestingly, this reward modulation did not persist into the delay period—precisely the time in which the dlPFC representation of reward was observed by Leon and Shadlen, and the period whose activity predicts an FEF neuron's ability to distinguish targets from distractors (Armstrong et al., 2009). This pattern of reward modulation contrasts starkly with the response properties of the DA neurons projecting to PFC, again emphasizing the role of DA-ergic activity as a modulator rather than a simple driver or inhibitor of prefrontal activity.

Spatially-specific representation of reward values in the FEF and rich DA-ergic inputs to this area raise the hypothesis that FEF DA could serve as a mechanism for reward-dependent selection of visual targets. Indeed, Soltani et al. pursued this idea and tested the behavioral effects of perturbing DA-ergic activity within the FEF of monkeys performing a saccadic choice task and simulated the effects using a biologically-plausible cortical network (Soltani et al., 2013). They found that manipulation of FEF activity either by blocking D1Rs or by stimulating D2Rs increased the tendency to choose targets in the RF of the affected site. These effects of DA manipulation could be described purely in terms of motor biases; however, DA manipulation also altered the influence of choice history, and hence reward history, on subsequent target choices. The effects of choice history were also differently altered by the two DA receptors: D1R manipulation decreased the tendency to repeat choices on subsequent trials, whereas the D2R manipulation increased that tendency. This altered impact of choice history indicates that manipulating FEF DA influences the value of saccadic targets based on prior reward experience. The network simulation results suggest that D1Rs influence target selection mainly through their effects on the strength of inputs to the FEF and on recurrent connectivity, whereas D2Rs influence the excitability of FEF output neurons. Altogether, these results reveal dissociable DA-ergic mechanisms influencing target selection in which D1Rs and D2Rs differentially alter saccadic target selection by virtue of their effects in different cortical layers (Noudoost and Moore, 2011c). The network model revealed that DA-ergic modulation of the afferents to the FEF could alter reward-dependent choice. Based on this model one might predict, for example, that after blocking D1Rs within the FEF, the form and time constant of reward integration would be altered such that the impact of previous rewards on current choices could be increased or decreased.

DA is a neuromodulator known to play a crucial role in reward-dependent behavior. Prefrontal neurons, which receive rich DA-ergic input from areas representing expected rewards, play a pivotal role in top-down modulation of cortical activity. Prefrontal DA (Noudoost and Moore, 2011b) and reward (Stănişor et al., 2013) can both modulate representation of targets within visual areas, mimicking some of the signatures of top-down visual attention. The questions of whether manipulation of PFC DA changes reward-dependent behavior, the degree to which signatures of attention and reward expectation in visual areas are dissociable, and whether DA-mediated PFC activity is the link for established behavioral interactions between attention and reward, remain to be answered.

## Norepinephrine

DA is not the only neuromodulator whose levels are critical for prefrontal function during cognitive tasks: NE also appears to be crucial to normal PFC activity. The PFC receives NE input from the locus coeruleus (Porrino and Goldman-Rakic, 1982; Levitt et al., 1984). The tonic firing of locus coeruleus NE neurons reflects arousal state, with low rates during slow wave sleep or drowsiness, moderate rates during waking, and high rates in response to acute stress. They also display phasic firing in response to behaviorally relevant stimuli during normal waking, but this phasic firing can extend to irrelevant distractors during fatigue or stress (Aston-Jones et al., 1999). Like the DA projections described above, NE inputs to PFC show a bilaminar targeting pattern (Morrison et al., 1982; Levitt et al., 1984; Lewis and Morrison, 1989). NE binds to high affinity alpha-2 adrenoreceptors, and to lower affinity alpha-1 and beta receptors (Molinoff, 1984). Alpha-2 receptors are found on dendritic spines in the superficial layers of PFC; although they can function both pre- and post-synaptically, their postsynaptic activity appears to underlie the benefits of alpha-2A agonists on working memory and other cognitive tasks (Arnsten and Cai, 1993; Wang et al., 2007). Like dopamine, there appears to be an optimal, intermediate level of NE signaling in PFC. The higher levels of NE associated with stress may impair PFC function through actions at the lower affinity alpha-1 receptors in the superficial layers (Arnsten et al., 1999; Birnbaum et al., 1999; Mao et al., 1999), and beta receptors localized on dendritic spines in the intermediate layers (Aoki et al., 1998; Ramos et al., 2005). Intracellularly, the actions of D1 (Vijayraghavan et al., 2007), alpha-2A (Wang et al., 2007), and beta1 receptors may converge on the cAMP signaling pathway (Gamo and Arnsten, 2011). Studies of the contributions of prefrontal NE to cognitive function led to the development of alpha-2A agonist guanfacine as a treatment for ADHD (Hunt et al., 1995; Taylor and Russo, 2001; Biederman et al., 2008; Gamo and Arnsten, 2011).

## Human studies of PFC catecholamines in normal and abnormal cognitive function

One of the reasons for focusing on prefrontal catecholamines—as opposed to, for example, prefrontal N-methyl-D-aspartate receptor (NMDA) or gamma-Aminobutyric acid (GABA) signaling, the proper functioning of which are certainly also vital to working memory and other prefrontal functions—is that these systems appear to be implicated in multiple disorders involving prefrontal dysfunction. Here we briefly canvas the literature linking prefrontal catecholamines to Parkinson's, schizophrenia, and ADHD, before turning to studies of their contribution to normal cognition in humans.

The loss of DA neurons in Parkinson's disease produces cognitive deficits in addition to the more outwardly apparent motor symptoms (Lees and Smith, 1983; Taylor et al., 1986; Morris et al., 1988; Owen et al., 1992, 1993; Postle et al., 1997). It seems likely that at least some of these cognitive effects are directly due to a loss of DA-ergic input to PFC, and can thus provide insight into the normal contribution of DA to these functions. Accordingly, multiple studies use the withdrawal of L-dopa or other dopaminergic medications in Parkinson's patients to evaluate the effect of reduced DA signaling on various cognitive tasks (Table 2). Results generally indicate impaired spatial working memory in the absence of sufficient DA (Lange et al., 1992; Mattay et al., 2002). They also confirm findings suggesting that increased prefrontal activity, measured with fMRI or blood flow, may reflect less efficient processing in these tasks, showing greater dlPFC activation in the hypo-DA-ergic state (Cools et al., 2002), and a correlation between increases in PFC activity and error rates on the working memory task (Mattay et al., 2002). Interestingly, in early Parkinson's disease patients DA loss is more pronounced in specific anatomical regions, with dramatic DA depletion in the putamen and dorsal caudate, while DA levels in the ventral striatum are relatively spared (Kish et al., 1988; Agid et al., 1993). These regions of the basal ganglia also differ in their prefrontal connectivity, the dorsal regions forming a circuit with dlPFC while the ventral striatum is connected to orbitofrontal cortex (Alexander et al., 1986). The consequences of this segregation and differential susceptibility to Parkinson's-induced DA losses can be seen in the effect of medication withdrawal on two tasks selected to differentially engage the dlPFC and the orbitofrontal cortex (Dias et al., 1996; Cools et al., 2001). Performance on task-set switching, which is thought to depend on dlPFC and parietal circuits, was impaired following medication withdrawal; in contrast, patients' performance on a reversal learning which depends upon orbitofrontal cortex actually improved when off of medication. This reinforces the notion of an optimal level of DA signaling: when disease-induced DA depletion affects circuits to different degrees, medication that increases DA globally and optimizes the level in one circuit may produce above-optimal levels in other areas, with corresponding behavioral deficits.

**Table 2:**
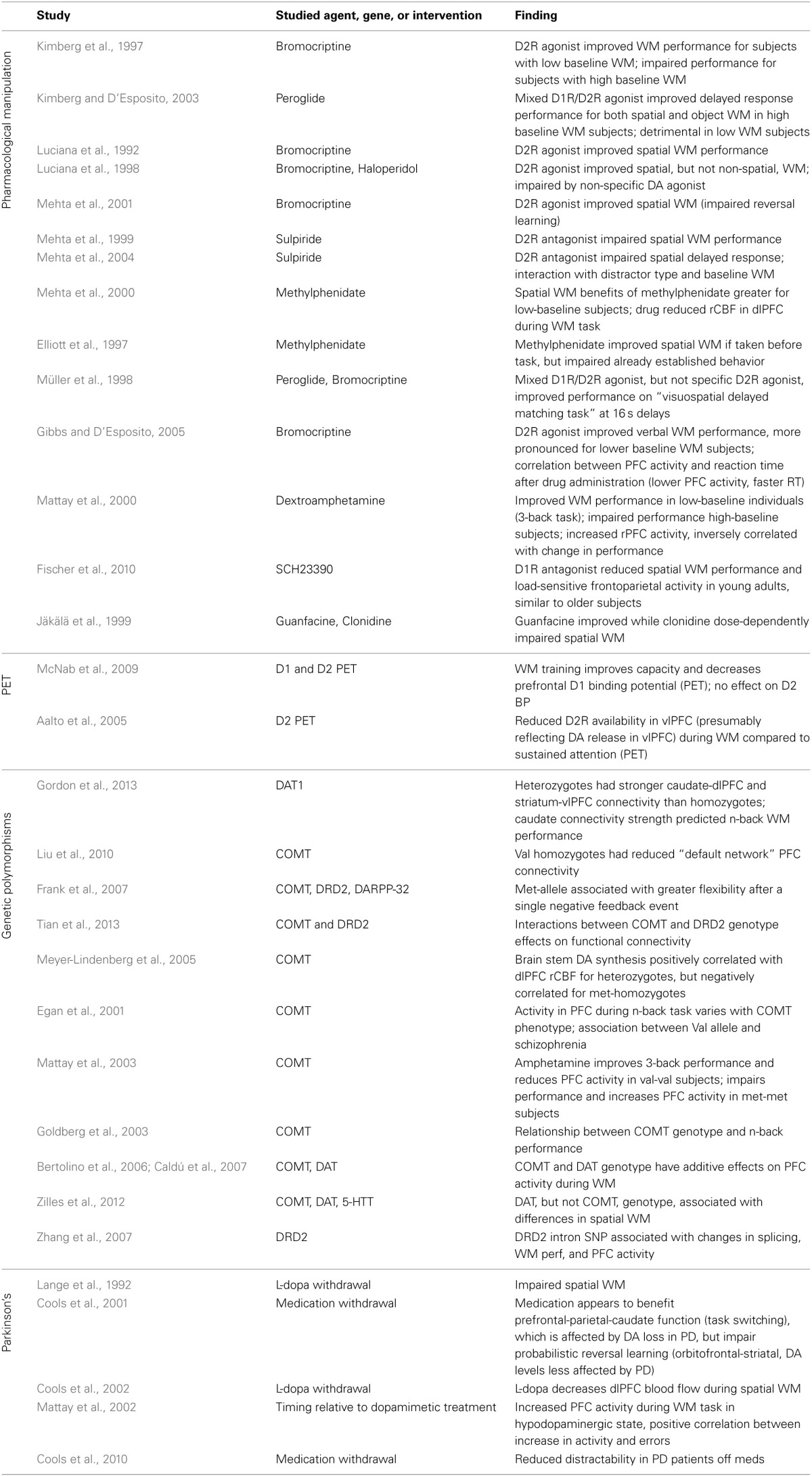
**Studies examining the contribution of prefrontal catecholamines to the behavioral and neural correlates of working memory in human subjects**.

Studies are grouped based on methodology: drug administration, PET, effects of genetic polymorphisms, and medication withdrawal in Parkinson's patients. Abbreviations and drug actions: DA, dopamine; bromocriptine, a D2 agonist; pergolide, an agonist for both D1 and D2 receptors; haloperidol, non-specific DA agonist; methylphenidate, amphetamine, and dextroamphetamine: stimulants producing an increase in PFC DA and NE release; sulpiride, D2 antagonist; guanfacine, alpha-2A agonist; clonidine, alpha-2 agonist; SCH23390, D1 receptor antagonist; DAT1, dopamine transporter gene; COMT, catechol-O-methyltransferase gene; 5-HTT, serotonin transporter gene; DARPP-32, dopamine- and cAMP-regulated neuronal phosphoprotein gene; DRD2, dopamine receptor D2 gene; WM, working memory; PET, positron emission tomography; dlPFC, dorsolateral prefrontal cortex.

DA is also implicated in the etiology of schizophrenia (origins of this idea reviewed in Baumeister and Francis, 2002). Although the “dopamine hypothesis” of schizophrenia has existed for decades, development of theoretical frameworks to link the pharmacological and neurobiological findings to the phenomenology of the disorder is ongoing, for example the aberrant salience theory of psychosis (Kapur, 2003; Kapur et al., 2005). Clinically effective antipsychotics appear to primarily target the D2 receptor (Seeman and Lee, 1975), and hyperstimulation of subcortical D2Rs is still considered a likely cause of the positive symptoms of the disorder; in contrast, a cortical, and specifically prefrontal, DA deficit may contribute to the cognitive symptoms (Abi-Dargham, 2004; Guillin et al., 2007). Associations have been found between schizophrenia and genetic variations in DA receptors (Glatt et al., 2003; Jönsson et al., 2004), and the COMT gene discussed below (Egan et al., 2001, reviewed in Harrison and Weinberger, 2005). COMT genotype has also been associated with the ability of antipsychotics to improve working memory performance (Weickert et al., 2004). (However, DA is not the only neurochemical system genetically linked to schizophrenia—see Mowry and Gratten, 2013). Schizophrenic patients display deficits in working memory tasks (Park and Holzman, 1992; Fleming et al., 1995; Morice and Delahunty, 1996; Keefe et al., 1997), and neurocognitive deficits have been shown to predict clinical outcomes (Green, 1996). Patients also show abnormal, typically excessive, PFC activation during these tasks (Manoach et al., 1999; Callicott, 2000; Barch et al., 2001; Perlstein et al., 2001). The laminar distribution of DA-ergic innervation of PFC appears altered (Akil et al., 1999), and there is some evidence for changes in prefrontal D1R density (Abi-Dargham et al., 2002, 2012)—although the absence of such effects in postmortem studies may indicate that expression levels are normalized by medication (Laruelle et al., 1990; Meador-Woodruff et al., 1997).

ADHD is one of the most common psychiatric disorders, affecting ~3–7% of the US population. Clinically, ADHD is characterized by inattention, impulsivity, and hyperactivity (American Psychiatric Association, 2013). In laboratory settings, ADHD patients' inattention and impulsivity lead to deficits in tasks measuring spatial attention (Friedman-Hill et al., 2010), working memory (Alderson et al., 2013), and oculomotor response inhibition (Rommelse et al., 2008; Goto et al., 2010). These cognitive tasks have long been linked to prefrontal function (D'Esposito and Postle, 1999; Miller, 2000). Given this link, it is unsurprising that patients with ADHD show structural and functional differences in prefrontal size, projection strength, resting connectivity, and activity during cognitive tasks (Seidman et al., 2005; Arnsten, 2006; Kieling et al., 2008). Several lines of evidence more specifically implicate prefrontal catecholamine function as an underlying cause and potential therapeutic target. Genetic linkage studies confirm potential contributions of both DA and NE to the disorder (reviewed in Gizer et al., 2009). Associated genes include DA receptors D1, D4, and D5 (Sunohara et al., 2000; Tahir et al., 2000; Kustanovich et al., 2004; Bobb et al., 2005; Mill et al., 2006; Wu et al., 2012), the DA transporter (DAT) (Durston et al., 2005; Mill et al., 2006), the NE transporter, the NE alpha-2A receptor (Xu et al., 2001; Roman et al., 2003), and DA beta-hydroxylase, an enzyme which coverts DA to NE (Daly et al., 1999; Roman et al., 2002; Kopecková et al., 2006). Many of the medications currently prescribed to treat ADHD alter catecholamine transmission (Arnsten, 2009). Stimulants such as amphetamine, lisdexamphetamine, and methylphenidrate block both DA and NE transporters. In rats, methylphenidrate (Ritalin®) has been shown to increase DA and NE release, particularly in the PFC (Berridge et al., 2006), and improve performance on a delayed alternation task used to assess prefrontal function in rodents. These performance benefits were blocked by co-administration of either an alpha-2A or D1R antagonist, neither of which impaired performance in isolation, suggesting that both DA-ergic and noradrenergic signaling contribute to the methylphenidate's cognitive effects (Arnsten and Dudley, 2005). Atomoxetine blocks the NE transporter, producing increases in both NE and DA in the PFC (Bymaster et al., 2002), while guanfacine is an alpha-2A receptor agonist.

Numerous studies have examined dopamine's contribution to cognitive performance by administering various DA agonists or antagonists to healthy volunteers (see Table 2). Unfortunately there is no D1R-selective drug available for use in humans; D1R effects have had to be inferred by comparing the effects of mixed agonists to those of D2R selective agents. A number of studies have reported the ability of DA-ergic drugs to alter performance on spatial working memory or delayed response tasks, although the studies' findings differ with respect to the relative contribution of D1Rs and D2Rs (Luciana et al., 1998; Müller et al., 1998) and whether the effects are limited to spatial working memory or apply to a broader range of memory and attention tasks (Luciana et al., 1998; Kimberg and D'Esposito, 2003). Some of this variability is probably attributable to an interaction between drug action and subjects' baseline DA-ergic tone (see discussion of the “inverted-U” action of DA above). Indeed, the action of these drugs in healthy volunteers has been shown to depend on their baseline working memory capacity (Kimberg and D'Esposito, 2003; Mattay et al., 2003). It may even depend on the subject's recent behavior: training on a working memory task, half an hour a day for 5 weeks, is sufficient to improve capacity measurements and decrease prefrontal D1R binding potential, suggesting DA receptor expression may be modulated by the demands of habitual tasks (McNab et al., 2009).

Genetic polymorphisms related to DA processing or signaling have also been linked to cognitive phenotypes, in both neurotypical and patient populations. One of the most extensively studied is a polymorphism in the catechol-O-methyltransferase (COMT) gene. COMT is an enzyme that breaks down DA following synaptic release; its activity is especially important for determining DA levels in the PFC, which has comparatively few DATs (Gogos et al., 1998). A common polymorphism producing a valine-to-methionine substitution alters enzyme activity: the Val-allele has higher enzymatic activity, presumably reducing prefrontal DA levels, while the Met-allele has lower activity, theoretically resulting in higher basal DA (Chen et al., 2004); however these presumed effects of COMT genotype on basal PFC DA levels have never been directly verified in humans. It should also be noted that the effects of many DA-related polymorphisms on working memory may be mediated by the striatum in addition to the PFC (Cools et al., 2008). Met-allele homozygotes show lower prefrontal activity during an n-back working memory task than heterozygotes, who in turn have lower prefrontal activation than Val-allele homozygotes (Egan et al., 2001). Amphetamine, which like other stimulants causes release of DA and NE in PFC (Kuczenski and Segal, 1992; Moghaddam et al., 1993; Berridge et al., 2006; Narendran et al., 2014), reduces prefrontal activity during the 3-back task in Val homozygotes, while increasing prefrontal activity and impairing performance for Met homozygotes on the same task (Mattay et al., 2003). These results are consistent with an inverted-U relationship between prefrontal DA levels and function, where Val homozygotes have slightly sub-optimal basal DA levels due to their increased enzymatic breakdown of DA, while Met homozygotes have higher basal DA levels, such that the additional DA release following amphetamine administration is detrimental to PFC function. Interestingly, Val-allele homozygotes show more perseverative errors on the Wisconsin card-sorting task, but no overall differences in working memory performance or other cognitive measures (Egan et al., 2001; Mattay et al., 2003; Zilles et al., 2012); this absence of baseline differences in working memory based on COMT genotype suggests compensatory changes in other aspects of DA signaling (although see Goldberg et al., 2003). The effects of COMT genotype on prefrontal activity during working memory have been shown to interact additively with another polymorphism, a variable number tandem repeat polymorphisms identified in the 3' untranslated region of the DAT gene (Bertolino et al., 2006; Caldú et al., 2007).

Performance in attention and working memory tasks is impaired in ADHD, Parkinson's disease, and schizophrenia, as well as under stress or in normal aging. Considering the evidence for a contribution of prefrontal catecholamines to these cognitive functions, imbalance in the prefrontal level of these neuromodulators has long been a suspected cause of the cognitive impairments observed in these disorders. More recently, genetic association studies have demonstrated links between prefrontal catecholamines and the etiology of these diseases, as well as how patients respond to treatment. Despite numerous studies examining the link between prefrontal DA or NE and cognitive function in these disorders, we are still far from treatments that fully restore cognitive function. This gap may be partly due to individual variation in the underlying pathology, but also partly as a result of our own incomplete understanding of the neural mechanisms underlying normal cognitive function. Even in cases where the mechanisms are well understood, clinically we lack the means to target specific anatomical or chemical subsets of neurons. However, basic research on the mechanisms of prefrontal function has produced some therapeutic advances, e.g., the introduction of guanfacine for the treatment of ADHD patients, and a more complete understanding of how prefrontal catecholamine signaling underlies cognition may produce further clinical applications.

## Conclusions and future directions

The link between prefrontal catecholamines and cognitive deficits in multiple neurological disorders makes understanding their role in prefrontal function particularly critical. While much progress has been made in elucidating the role of prefrontal catecholamines' role in cognitive function, crucial questions still remain. Is the effect of prefrontal DA mediated entirely via reward expectation, or do basal PFC DA levels modulate working memory and attention performance in a manner dissociable from upcoming rewards? Do PFC DA levels fluctuate significantly over the course of attention and working memory tasks, and do these fast changes in DA signaling contribute to behavioral performance? Although the true “neural mechanism” of working memory maintenance or covert attentional deployment is the pattern of task-related neural activity, driven by spatially tuned glutamatergic and GABA-ergic responses, these population dynamics are enabled by appropriate DA and NE “tone” within these prefrontal circuits; whether more temporally or spatially localized changes in catecholamine signaling also contribute to task performance (Chadderdon and Sporns, 2006) remains uncertain. More reliable, temporally precise and continuous measures of local DA levels would be an important first step in addressing these questions.

### Conflict of interest statement

The authors declare that the research was conducted in the absence of any commercial or financial relationships that could be construed as a potential conflict of interest.
