# Corrigendum: “The role of prefrontal catecholamines in attention and working memory”

**DOI:** 10.3389/fncir.2014.00142

**Published:** 2014-12-02

**Authors:** Behrad Noudoost, Kelsey L. Clark

**Affiliations:** ^1^Department of Cell Biology and Neuroscience, Montana State UniversityBozeman, MT, USA

**Keywords:** dopamine, reward, top-down control, frontal eye field, V4

On Page 4, second column, first paragraph, the sentence currently reading (errors in bold):

“One group of PFC neurons, which included all the modulated narrow-spiking, putatively inhibitory neurons, was inhibited by DA; these showed short onset latency of DA effects (~ 10 **ms**), with no change in signal-to-noise ratio (SNR) or inter-trial variability. A second set of prefrontal neurons was excited by DA application, displaying an increase in SNR and decrease in inter-trial variability; this effect was slower (~ 200 **ms**) and observed only in broad-spiking, putatively pyramidal neurons.”

Both instances of “ms” (milliseconds) in this sentence should be changed to seconds, “s.” Correct text will read:

“One group of PFC neurons, which included all the modulated narrow-spiking, putatively inhibitory neurons, was inhibited by DA; these showed short onset latency of DA effects (~ 10 s), with no change in signal-to-noise ratio (SNR) or inter-trial variability. A second set of prefrontal neurons was excited by DA application, displaying an increase in SNR and decrease in inter-trial variability; this effect was slower (~ 200 s) and observed only in broad-spiking, putatively pyramidal neurons.”

## Conflict of interest statement

The authors declare that the research was conducted in the absence of any commercial or financial relationships that could be construed as a potential conflict of interest.

